# Laser Ablation of Aluminum Near the Critical Regime: A Computational Gas-Dynamical Model with Temperature-Dependent Physical Parameters

**DOI:** 10.3390/mi12030300

**Published:** 2021-03-12

**Authors:** Jacopo Terragni, Antonio Miotello

**Affiliations:** Department of Physics, University of Trento, Via Sommarive 14, 38123 Povo, Italy; antonio.miotello@unitn.it

**Keywords:** nanosecond laser ablation, gas-dynamics, multi-phase modeling, critical thermodynamic regime, aluminum

## Abstract

The complexity of the phenomena simultaneously occurring, from the very first instants of high-power laser pulse interaction with the target up to the phase explosion, along with the strong changes in chemical-physical properties of matter, makes modeling laser ablation a hard task, especially near the thermodynamic critical regime. In this work, we report a computational model of an aluminum target irradiated in vacuum by a gaussian-shaped pulse of 20 ns duration, with a peak intensity of the order of $GW/cm{}^{2}$. This continuum model covers laser energy deposition and temperature evolution in the irradiated target, along with the mass removal mechanism involved, and the vaporized material expansion. Aluminum was considered to be a case study due to the vast literature on the temperature dependence of its thermodynamic, optical, and transport properties that were used to estimate time-dependent values of surface-vapor quantities (vapor pressure, vapor density, vapor and surface temperature) and vapor gas-dynamical quantities (density, velocity, pressure) as it expands into vacuum. Very favorable agreement is reported with experimental data regarding: mass removal and crater depth due to vaporization, generated recoil momentum, and vapor flow velocity expansion.

## 1. Introduction

The application of the laser ablation (LA) technique covers a great variety of scientific and technological fields: Pulsed Laser Deposition (PLD) [1], nanoparticles production [2], Laser Induced Breakdown Spectroscopy (LIBS) for chemical analysis of solid materials [3], high-precision artwork restoration [4], investigation of the conditions for inertial confinement fusion [5]. Despite being so widely used, LA is far from being completely understood. Even if this technique can be simply summarized by the interaction of an intense laser pulse with a target and the consequent material emission, there is a wide variety of physical phenomena occurring in this process, which are strongly dependent on laser pulse features, target material composition, and ambient atmosphere. In recent years, the possibility of extending such a technique to the Space technology context, in connection with both the space debris issue [6] and Laser Ablation Propulsion (LAP) as a new propulsive concept [7], has brought back the attention to the fundamental aspects of LA, in particular to the dynamics of the emitted material. As a consequence, in this work we focused on LA, in vacuum, of an aluminum target generated by a Gaussian-shaped laser pulse of 20 ns duration (FWHM) with a peak intensity of the order of $GW/cm{}^{2}$. The choice of this target material naturally arises by considering that a consistent part of space debris is made of aluminum [8] and that aluminum is also widely used in LAP, both as a propulsion performance standard [9] and as a case study for propulsion optimization [10].

It is now widely accepted that two main thermal mechanisms contribute to LA, namely vaporization and phase-explosion [11,12,13].

Modeling vaporization regime has been widely investigated since the very first steps of this technique [14]. The rate of outgoing vapor from the surface is generally modeled via Hertz-Knudsen equation, as used in [15,16]. Despite being widely used, even in recent works [17], this expression is not derived from a mass conservation relation across the target surface and so, here, we choose to follow the surface recession description as proposed by D. Autrique et al. [18], in which surface recession is linked to vapor velocity beyond the Knudsen layer (KL) via mass continuity equation (see Section 2.3).

KL plays a key-role in the vapor expansion since it is in this thin layer (few mean free paths) that vapor particles reach traslational equilibrium and get a well-defined flow velocity [19]. Relations among the gas-dynamical quantities at the surface and beyond KL were analytically derived by C.J. Knight [20], obtaining the so-called KL jump conditions. In 1990, R. Kelly in [21] ended up to the same results considering an unsteady adiabatic expansion (UAE) for the outgoing vapor.

However, the plasma-shielding effect is not considered. This phenomenon consists of the partial or total absorption of the laser light directed towards the target by the outgoing material, reducing the actual amount of optical energy reaching the target and partially ionizing the vapor, which turns to be the so-called plume. If this absorption process is taken into account, then the adiabatic hypothesis does not hold and KL relations should not be used [17,22].

In our work, the plasma-shielding effect is not also covered in the model, then the whole energy per unit area (Fluence, see Section 2.1) brought by the pulse reaches the target. If one aims to model the actual plume expansion, the starting point is to consider the plume as a low temperature plasma (LTP), as deeply reviewed in [23].

Phase-explosion (sometimes called explosive boiling) is the physical phenomenon occurring in a super-heated metastable liquid where the rate of homogeneous nucleation of vapor bubbles increases dramatically and the hot region breaks down into vapor plus liquid droplets. Such phenomenon occurs exclusively in proximity of the critical temperature ($T∼0.9T_{c}$) because only in this temperature regime it is possible to have large density fluctuations that result in vapor bubbles generation. This concept was firstly pioneered by M.M. Martynyuk in 1976 [15] and then, notwithstanding the unfavourable time-scale (1 ns to 100 ns) advocated in that paper, successfully proved by A. Miotello and R. Kelly in 1995 [24] as the most efficient mechanism in the ablation process when looking at thermal processes.

A computational approach to this ablative regime has been considered only in recent years [25,26] and it is not covered in this work. Anyway, few considerations about phase-explosion can be extracted from our model and they are briefly presented in Section 5.

Since the target temperature ranges from room temperature up to its critical one, any attempt to correctly model LA should take into account the strong variation occurring in the thermophysical, optical, and transport properties of the target material induced by the laser pulse, as importantly highlighted by D. Marla et al. in [27].

In the present work, a special effort is devoted to the use of temperature-dependent thermodynamic parameters up to extreme thermodynamic conditions, justified as far as possible at an experimental level and possibly on the basis of theoretical models with the aim of showing how gas-dynamic processes evolve under conditions also near the critical temperature.

The temperature dependence of these physical quantities, obtained from the available experimental data along with semi-empirical laws, is presented in Section 3.

A continuum model for ns-LA of Al with temperature dependent physical properties is used because the time-scale of the laser pulse is around nanoseconds and so the optical energy in metals can be regarded as being instantaneously turned into heat (electron-phonon coupling can take from fractions of a picosecond up to tens of picoseconds [28]).

A continuum model of ns-LA generally leads to the solution of a set of interconnected partial differential equations describing the laser energy deposition and evolution of temperature in the irradiated target, along with the mass removal mechanism involved, and the expansion of the vaporized plume. The continuum model used in this work is presented in Section 2, while the numerical methods used to perform calculations are presented in Section 4.

The main results of this work are presented in Section 5. The most relevant ones regard the actual computation of the gas-dynamical quantities of the vapor expansion in the external vacuum region, going beyond with respect to the work proposed by Marla et al. in [22], where the emitted vapor was considered to be a thin uniform region of constant particle density close to the target. The calculated vapor expansion has been found in very good agreement with experimental measures performed in the same conditions simulated in this work, as discussed in Section 5.1. As far as our knowledge goes, this is the first work based on temperature-dependent physical parameters in which the computed gas-dynamic expansion from LA has been positively compared to experimental measures [29].

Moreover, the temperature dependence of the LA modeling has been investigated by comparison with simulation with fixed physical parameters for each phase of matter (see Section 5). We found how the temperature dependence of the parameters of the ablated target is of capital importance in studying the surface modification and resolidification process.

Finally, we developed a robust numerical scheme for the gas-dynamic process based on the numerical Rusanov’s Flux (see Section 4) that well behaved in managing time-dependent boundary conditions and that could be used in other attempts to simulate LA with temperature-dependent physical parameters.

## 2. Modeling Laser Ablation

The laser pulse impinging on the target is here described by a Gaussian-shaped temporal intensity profile ($I_{\mathrm{laser}}\left(t\right)$) of 20 ns duration (FWHM):

(1) $$I_{\mathrm{laser}(t)}=I_{\max}\exp\left[-\frac{\log\left(16\right){\left(t-\tau /2\right)}^{2}}{{\mathrm{FWHM}}^{2}}\right],$$

where τ and *t* are respectively the total pulse duration and the time.

The peak intensity of the laser pulse can be restated in a more practical way with respect to the laser fluence (*F*) (i.e., the amount of optical energy per unit area) as

(2) $$I_{\max}=\frac{2F}{\mathrm{FWHM}}\sqrt{\frac{\log(2)}{\pi }}.$$

### 2.1. Thermal Processes

**Figure 1:**
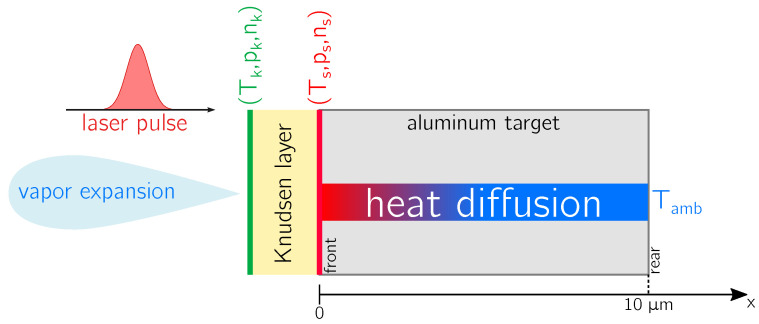
Schematic (not quantitative) representation of the laser pulse, the irradiated aluminum target, and the expanding vapor. The laser pulse impinges on the front surface of the target and the absorbed laser optical energy is converted into heat which diffuses within the target. The target is taken to be thick enough so that its rear surface can be still considered at room temperature. The role of the Knudsen layer and the related physical quantities are emphasized in the figure.

In metals, light is mainly absorbed by free electrons in a quasi-linear regime. This allows the application of the Beer-Lambert law to describe the laser intensity propagation inside the target, which reads

(3) $$I\left(x,t\right)=I\left(0,t\right)e^{-\alpha (T)x},$$

where α(T) is the temperature-dependent absorption coefficient, *x* is the spatial coordinate as represented in Figure 1, and x=0 is the instantaneous surface position.

Once the optical energy has been converted into material internal energy at the typical time-scale of ∼$10^{-13}$ s, the heat diffusion takes place. The absorption process acts as a source term S(x,t) in the governing 1D conservation law for the internal energy density

(4) $$\frac{\partial q(x,t)}{\partial t}-v_{s}\frac{\partial q(x,t)}{\partial x}+\frac{\partial f(x,t)}{\partial x}-S\left(x,t\right)=0,$$

with $v_{s}$ being the surface recession velocity due to vaporization [30] and

(5) $$S\left(x,t\right)=\alpha \left(T\right)I\left(x,t\right)=\alpha \left(T\right)\left[1-R(T_{s})\right]I\left(0,t\right)e^{-\alpha (T)x},$$

where $R(T_{s})$ is the target reflectivity which depends on the surface temperature $T_{s}$.

The heat flux f(x,t) inside the target can be described by the classical Fourier law, given by

(6) $$f\left(x,t\right)=-\kappa \left(T\right)\frac{\partial T}{\partial x},$$

with κ(T) being the thermal conductivity. To take into account the solid-liquid phase transition generally occurring in LA, the temperature dependence of the internal energy density is given by

(7) $$q\left(T\right)=\left\{\begin{array}{cc} \rho \left(T\right)c_{p}\left(T\right)\left(T-T_{m}\right) & \mathrm{if}\;T<T_{m}\\ \rho \left(T\right)c_{p}\left(T\right)\left(T-T_{m}\right)+\rho \left(T\right)\Delta h_{m} & \mathrm{if}\;T>T_{m} \end{array}\right.$$

where ρ(T) is the target density, $c_{p}\left(T\right)$ is the specific heat at constant pressure, $T_{m}$ is the melting point and $\Delta h_{m}$ is the latent heat of melting.

Boundary conditions of the heat diffusion process are provided by the following relations (see Figure 1):

(8a) $$\begin{array}{cc} \mathrm{IC}: & \;T\left(x,0\right)=T_{\mathrm{amb}}; \end{array}$$

(8b) $$\begin{array}{cc} \mathrm{BC}1: & \;f\left(x_{\mathrm{front}}=0,t\right)=-\kappa \left(T\right)\frac{\partial T(0,t)}{\partial x}=\Delta h_{v}\left(T\right)\rho \left(T\right)v_{s}; \end{array}$$

(8c) $$\begin{array}{cc} \mathrm{BC}2: & \;T\left(x_{\mathrm{rear}},t\right)=T_{\mathrm{amb}}. \end{array}$$

The initial condition (8a) sets the target initial temperature to the room temperature $T_{\mathrm{amb}}$. The first boundary condition (8b) accounts for the loss of internal energy density due to surface vaporization ($\Delta h_{v}$ is the latent heat of vaporization) in the front part of the target. The second one (8c) is established since the heat diffusion process does not reach the rear part of the target. To ensure the validity of this last condition, we consider here, without any loss of generality, a target of thickness 10 μm and, consequently, $x_{\mathrm{rear}}=10$μm. The solution of (4) (see IV A), along with (5)–(8), returns the temperature within the target (T(x,t)). In particular, its time-dependent surface value ($T_{s}\equiv T\left(0,t\right)$) is the starting point to model the vaporization phenomenon, as discussed in the following Section 2.2.

### 2.2. Vaporization

Once $T_{s}$ has been computed, the saturated vapor pressure ($p_{s}$) acting on the target surface can be obtained via the Clausius-Clapeyron relation:

(9) $$\frac{dp_{s}}{dT_{s}}=\frac{\Delta h_{v}\left(T_{s}\right)}{T_{s}\left[v_{\mathrm{vap}}-v^{*}\right]},$$

where $v_{\mathrm{vap}}$ is the molar volume of the vapor phase and $v^{*}$ is the molar volume of the condensed phase (solid or liquid) of the target surface, which is generally negligible with respect to $v_{\mathrm{vap}}$.

Here we assumed the saturated vapor to behave ideally. Actually, real gases are well-described by the ideal gas law at high temperature and low density. The saturated vapor produced in LA satisfies only the former of the previous conditions and so it should in principle deviate from being ideal. Despite of this, the ideal gas approximation relies on two considerations:

1. the necessary temperatures to attain efficient LA of metals (our situation) are very high due to their high $T_{c}$. This implies that the interatomic potential becomes certainly negligible with respect to the atomic kinetic energy, and precisely this contributes to justify the assumption of randomly moving particle via perfectly elastic collision which the ideal gas is based on;
2. modeling the vapor expansion in vacuum, using the Euler Equations (15), requires the Knudsen layer to be taken into account. Now all the quantitative relations connected to it and available in the literature, see for example [21], have been calculated under the ideal gas approximation, which is therefore normally used in the literature (see also [31]). This is the reason we have consistently used the ideal gas approximation throughout the text.

Due to previous considerations, $p_{s}$ is given by

(10) $$p_{s}\left(T_{s}\right)=p_{0}\exp\left[\frac{\Delta h_{v}\left(T_{s}\right)\left(T_{s}-T_{b}\right)}{RT_{s}T_{b}}\right],$$

where *R* is the gas constant, $p_{0}$ is the ambient pressure, and $T_{b}$ is the normal boiling point.

Consequently, the vapor number density can be obtained as

(11) $$n_{s}\left(T_{s}\right)=\frac{p_{s}}{k_{B}T_{s}}.$$

### 2.3. Knudsen Layer and Vapor Expansion

Aluminum atoms are ejected from the surface due to vaporization and their motion is supposed to be ballistic with main direction orthogonal to the target. Collisions occur after the surface detachment and transform atomic singular ballistic velocity into a collective flow velocity (*u*), which is consequently the centre of the atomic Maxwellian velocity distribution function, as stated in [30]. This spatial region in which the translation equilibrium is established is known as Knudsen Layer (KL) and it is schematically (not quantitatively) shown in Figure 1.

Moreover, the ablative regime here considered leads to an unsteady adiabatic expansion (UAE) where the flow velocity of the outgoing vapor is equal to the speed of sound [21] (i.e., the vapor flow has unitary Mach number)

(12) $$u_{k}={\left(\frac{\gamma k_{B}T_{k}}{m}\right)}^{1/2}={\left(\frac{5k_{B}T_{k}}{3m}\right)}^{1/2}$$

where the subscript *k* refers to the outer boundary of the KL and γ=5/3 is the specific heat ratio for a monoatomic ideal gas. Keeping ideal the vapor state equation, then its temperature, density and pressure jumps across the KL can be computed:

(13) $$T_{k}=0.669T_{s};\;\rho_{k}=0.308\rho_{s}.$$

Moreover, the mass conservation across the KL region provides the continuity equation

(14) $$\rho_{s}v_{s}=\rho_{k}u_{k}$$

which let us compute the surface recession velocity $v_{s}$ due to vaporization, where $\rho_{s}$ is the target density at the surface. The right hand side of (14) depends on $T_{s}$ via (12) and (13). As described in Section 2.1, $T_{s}$ is a time-dependent value and then $v_{s}$, as shown in Figure 4, panels (e) and (f), is also time-dependent.

The gas-dynamic of the expanding vapor beyond the KL is governed by Euler’s equations for compressible media [32]:

(15a) $$\frac{\partial \rho }{\partial t}+\frac{\partial \rho u}{\partial x}=0$$

(15b) $$\frac{\partial (\rho u)}{\partial t}+\frac{\partial }{\partial x}\left(\rho u^{2}+p\right)=0$$

(15c) $$\frac{\partial E}{\partial t}+\frac{\partial }{\partial x}\left(E+p\right)u=0$$

with $E=\left(\frac{1}{2}\rho u^{2}+e\right)$ being the vapor total energy density given by the sum of the kinetic energy density and the internal energy density (*e*). The solution of the PDE set (15) is provided by the closure relation $e=\frac{p}{\left(\gamma -1\right)\rho }$, along with the boundary conditions $u_{k}$, $p_{k}$, $\rho_{k}$. Here the spatial coordinate *x* must be intended perpendicular to the target surface and directed in the outgoing direction as depicted for the vapor expansion in Figure 1 with the origin set on the outer boundary of the Knudsen layer.

The choice for continuum equations to describe the vapor expansion is justified both by presence of the Knudsen layer (see [30]) as well as for its extension (a few atomic mean free path, see [30], of the order of few nm). On the contrary, note that the integration domain of the gas-dynamic equations is, in the present case, of ∼ mm order (see results reported in Figures 7 and 8).

## 3. Aluminum Properties

In most applications in which ns-LA is involved, materials explore a large range of temperature, starting from room temperature up to the critical one.

Due to the strong difficulties connected with the experimental evaluation of the thermodynamic and optical quantities in the high temperature regime, especially beyond the boiling point, theoretical and semi-empirical models are required to estimate the relevant physical properties. The parameters used in these models are listed in Table 1, Table 2 and Table 3.

In this section, thermophysical, optical and transport properties of aluminum will be investigated. For values in the solid state we refer to [33].

### 3.1. Critical Temperature

The starting point of any attempt to estimate aluminum properties beyond the boiling point, is to establish its critical temperature $T_{c}$. Here we follow the method proposed by Blairs et al. in [34] in which $T_{c}$ is related to the surface tension ($\sigma_{m}$) [35] and the liquid molar volume ($v_{m}$) [36] at the melting point

(16) $$T_{c}=\sigma_{m}{\left(\frac{v_{m}m}{{\left(Cv_{m}\right)}^{5/6}-q}\right)}^{4},$$

where the empirical fit parameters values are q= −1.0459 × 10^−25^, m= 8.9733 × 10^−19^ and C=1.484±0.025.

### 3.2. Density

The aluminum density in the solid state, as well as in the liquid state up to $T_{b}$, has been deeply studied both from a theoretical and an experimental point of view, but its behaviour from $T_{b}$ to $T_{c}$ is far from being definitely understood. Consequently, we decided to model the aluminum liquid density as a power series, with universal exponents and four material-dependent coefficients (Table 2), following the approach proposed in [31]:

(17) $$\rho_{l}\left(T\right)=\rho_{c}\left[1+D_{0}\Delta T+C_{1}{\left(\Delta T\right)}^{\beta_{c}}+D_{1}{\left(\Delta T\right)}^{1-\alpha_{c}}+C_{2}{\left(\Delta T\right)}^{\beta_{c}+\Delta_{c}}\right]$$

where $\Delta T=\frac{T_{c}-T}{T_{c}}$ and $\rho_{c}$ is the critical density (Table 1). It is worth noticing that independently from the choice of the coefficients, Equation (17) is consistent with the fact that the liquid density tends to the critical density $\rho_{c}$ when *T* tends to $T_{c}$.

### 3.3. Specific Heat at Constant Pressure

The isobaric specific heat in the liquid phase, from $T_{m}$ to $T_{c}$ is given by the semi-empirical formula [37]:

(18) $$c_{p,l}=c_{p,l}\left(T_{m}\right){\left(\frac{\Delta T}{\Delta T_{m}}\right)}^{-0.24}.$$

The value of $c_{p,l}\left(T_{m}\right)$ is 1070 J/(kgK) [38].

### 3.4. Enthalpy of Vaporization

Concerning the enthalpy of vaporization $\Delta h_{v}\left(T\right)$, we make use of the well-known Watson’s formula [39]:

(19) $$\Delta h_{v}\left(T\right)=\Delta h_{v}\left(T_{b}\right){\left(\frac{\Delta T}{\Delta T_{0}}\right)}^{0.38},$$

which has been validated for metals from $T_{b}$ to $T_{c}$ [40].

### 3.5. Transport Properties

As widely discussed by Marla et al. [27] aluminum can be considered to be a free electron metal in its liquid state up to the critical temperature. Consequently, thermal conductivity is related to the electrical conductivity via the Wiedermann-Franz law [41]:

(20) $$K\left(T\right)=\frac{\pi^{2}}{3}{\left(\frac{k_{B}}{e}\right)}^{2}T\sigma \left(T\right).$$

Experimental values for electrical conductivity are taken from [38].

### 3.6. Optical Properties

Optical properties play a key role in ns-LA since they determine the optical energy conversion into the internal energy of the target. In particular, the temperature dependence of both absorption coefficient (α) and reflectivity (*R*) exhibit a large variation over the full range from $T_{\mathrm{amb}}$ to $T_{c}$, and they can be both related to the complex dielectric function (ϵ).

In this work the dielectric function is computed via the Drude two-critical point model [42]:

(21) $$\epsilon \left(\omega \right)=\epsilon_{\infty }-\frac{\omega_{p}^{2}}{\omega^{2}+i\nu_{e}\omega }+\sum_{n=1}^{2}A_{n}\Omega_{n}\left(\frac{e^{i\phi_{n}}}{\Omega_{n}-\omega -i\Gamma_{n}}+\frac{e^{-i\phi_{n}}}{\Omega_{n}+\omega +i\Gamma_{n}}\right),$$

where all parametric values are listed in Table 3.

From the complex dielectric function, the real $n_{R}$ and the imaginary $n_{I}$ part of the refraction index can be obtained:

(22) $$\epsilon =\epsilon_{R}+i\epsilon_{I}={\left(n_{R}+in_{I}\right)}^{2}.$$

Then temperature dependence of the reflectivity (23) and the absorption coefficient (24) can be obtained from the Fresnel formulas:

(23) $$R\left(T\right)=\frac{{\left[n_{R}\left(T\right)-1\right]}^{2}+n_{I}^{2}\left(T\right)}{{\left[n_{R}\left(T\right)+1\right]}^{2}+n_{I}^{2}\left(T\right)},$$

(24) $$\alpha \left(T\right)=\frac{4\pi }{\lambda }n_{I}\left(T\right)$$

where λ is the laser irradiating wavelength. In this work we considered an excimer KrF laser with λ=248 nm.

## 4. Numerical Methods

**Figure 2:**
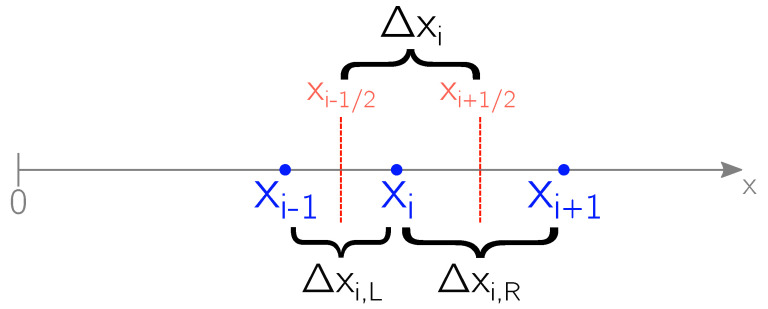
FV discretization of the spatial coordinate x involved in the numerical solution of (4) ÷ (8c) via the numerical scheme shown in (25).

To calculate the temperature T(x,t) within the irradiated target, the numerical integration of the set of Equations (4) ÷ (8c), describing the heat diffusion process, has been performed via a Finite Volume (FV) scheme. Since the thermophysical parameters of aluminum are taken to be temperature dependent in this model, the numerical approximation here used for the differentiation is Forward in Time Centered in Space (FTCS) [43]. The target has been subdivided into discrete volumes of increasing size moving from the front to the rear surface and the schematic representation of volumes and their boundaries is shown in Figure 2.

The scheme for the evolved temperature within the *i*-th volume of length $\Delta x_{i}$ at time instant n+1 ($T_{i}^{n}+1$) is given by

(25) $$\begin{array}{cc} T_{i}^{n+1}= & \;T_{i}^{n}+\frac{\Delta t}{\Delta x_{i}}\frac{v_{\mathrm{rec}}}{2}\left(T_{i+1}^{n}-T_{i-1}^{n}\right)+\frac{\Delta t}{\Delta x_{i}}\frac{1}{\rho_{i}^{n}c_{p,i}^{n}}\left(k_{i+1/2}\frac{T_{i+1}^{n}-T_{i}^{n}}{\Delta x_{i,R}}-k_{i-1/2}\frac{T_{i}^{n}-T_{i-1}^{n}}{\Delta x_{i,L}}\right)\\ \; & +\frac{\Delta t}{2\rho_{i}^{n}}\frac{\alpha_{i}^{n}}{c_{p,i}^{n}}\left(I_{i}^{n}+I_{i}^{n+1}\right) \end{array}$$

where $\kappa_{i\pm 1/2}\equiv \frac{\kappa_{i}^{n}+\kappa_{i\pm 1}^{n}}{2}$ is the approximated thermal conductivity at the boundaries of the *i*-th volume at the time step *n* and $v_{\mathrm{rec}}\equiv v_{s}\left(T_{s}^{n}\right)$ is the surface recession velocity at the time step n. The other parameters present in (25) maintain the definition given in Section 2.1 and they are related to the *i*-th volume at the time step *n*. $\Delta x_{i,L}=x_{i}-x_{i-1}$ and $\Delta x_{i,R}=x_{i+1}-x_{i}$, as shown in Figure 2.

The stability of the scheme is governed by the so-called parabolic time-step (Δt) restriction, which here reads:

(26) $$\Delta t\leq \min_{j}\left[\frac{\Delta x_{j}^{2}}{2{\tilde{\lambda }}_{j}}\right]$$

where $\Delta x_{j}$ is the *j*-th volume length and $\tilde{\lambda }$ is its thermal diffusivity.

The numerical resolution of the Euler equations has been also performed via a FV scheme [32] with volumes of increasing size moving from the outer boundary of the KL along the vapor expansion of Figure 1. Figure 2 is still a correct representation of the considered volumes and their boundaries. The standard semi-discrete conservative formulation of the Euler equations is given by

(27) $${\vec{U}}_{t}+\vec{F}{\left(\vec{U}\right)}_{x}=0\;\to \;\frac{d{\vec{U}}_{i}}{dt}=-\frac{1}{\Delta x_{i}}\left({\vec{F}}_{i+1/2}-{\vec{F}}_{i-1/2}\right),$$

where $U=\left[\rho ,\rho u,E+\rho u^{2}\right]$ is the vector of the conserved quantities as stated in Equation (15). The numerical flux of the conserved gas-dynamic quantities here chosen is the one proposed by Rusanov [44] with piecewise linear reconstruction (TVD—Van Albada’s slope limiter [32]) at the volume boundaries:

(28) $${\vec{F}}_{i+1/2}=\frac{1}{2}\left({\vec{F}}_{L}+{\vec{F}}_{R}\right)-\frac{1}{2}S^{+}\left({\vec{U}}_{i+1/2}^{+}-{\vec{U}}_{i+1/2}^{-}\right),$$

where

(29) $${\vec{F}}_{L}=\vec{F}\left({\vec{U}}_{i+1/2}^{-}\right);\;{\vec{F}}_{R}=\vec{F}\left({\vec{U}}_{i+1/2}^{+}\right);$$

and

(30) $$S^{+}=\max\left[|u_{L}|+a_{L},\left|u_{R}\right|+a_{R}\right].$$

Here ${\vec{F}}_{L}$, ${\vec{F}}_{L}$ are the analytical fluxes evaluated on the left side $\left({\vec{U}}_{i+1/2}^{-}\right)$ and the right side $\left({\vec{U}}_{i+1/2}^{+}\right)$ of the right boundary of the volume (*i*) in which the calculation is performed; $u_{L}$, $a_{L}$, and $u_{R}$, $a_{R}$ are the flow velocities and sound velocities at the same boundary, and they are estimated via Roe’s averages [32].

The sound velocity is computed with the ideal gas condition

(31) $$a=\sqrt{\frac{\gamma RT}{M}}=\sqrt{\frac{\gamma p}{\rho }},$$

where *M* is the molar mass of the considered gas atom and γ=5/3 is the specific heat ratio of the monoatomic ideal gas.

Time integration of (28) has been performed via second order Runge-Kutta method (RK2). The stability of the scheme is guaranteed by the usual Courant-Friedrich-Levy (CFL) condition [32]:

(32) $$\mathrm{CFL}=\frac{\Delta t}{\Delta x_{j}}\max\left(S_{j}^{+}\right)<1\;\forall j.$$

The time-step of the full computation has been kept adaptive with respect to the parabolic restriction checking at every new iteration that the CFL condition was fulfilled.

## 5. Results and Discussion

Here we show a few plots of the most relevant numerical results we obtained with the model presented in Section 4.

**Figure 3:**
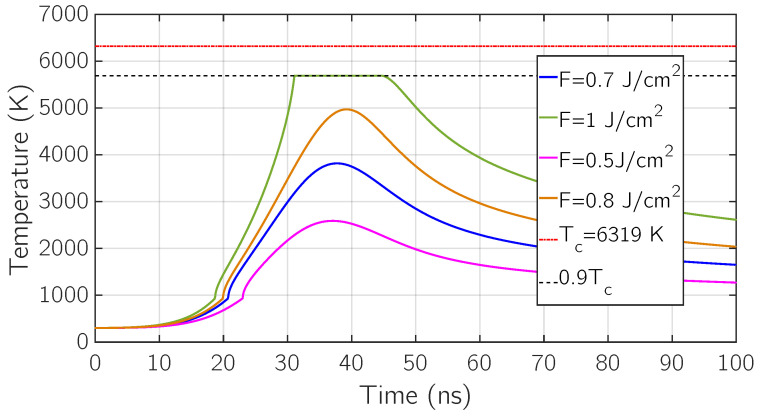
Surface temperature over time for different fluence values *F* = 0.7, 0.8, 1 J/cm^2^ (blue, orange, green) and *F* = 0.5 J/cm^2^ (pink).

We start by considering the heat-up and thermal diffusion process inside the target material. In Figure 3 the surface temperature with respect to time is depicted for four different fluences: *F* = 0.5 J/cm^2^, *F* = 0.7 J/cm^2^, *F* = 0.8 J/cm^2^, *F* = 1 J/cm^2^.

First of all, we notice that higher the fluence, higher the maximum value of the surface temperature, since a larger amount of energy is released by the laser pulse and absorbed in the first layers of the target.

Moreover, looking at the fluences *F* = 0.5 J/cm^2^, 0.7 J/cm^2^ and 0.8 J/cm^2^, it could be noticed that the time positions of the surface temperature maxima are slightly shifted to the right and that they are located between 30 ns and 40 ns, a few nanoseconds after the laser intensity maximum which is set at exactly 30 ns, centre of the Gaussian-shaped laser pulse $I_{\mathrm{laser}}\left(t\right)$.

This can be explained in terms of balance of energy fluxes at the surface. The first one is given by the laser intensity, which is the incoming energy flux from the external region through the surface according to (1) and it is a surface heating flux. The second one is given by the heat flux generated by the temperature gradient between the high-temperature surface layers and the low-temperature inner layers, which is the outgoing energy flux from the surface layer to the target according to (6) and the third one is the energy flux dispersed due to surface vaporization. The last two are surface cooling fluxes.

Focusing on the curve obtained at *F* = 0.5 J/cm^2^, it is worth to notice that such amount of energy per unit area is not enough to get the sample into the boiling regime, since the maximum value of surface temperature is just a little below the boiling temperature $T_{b}$.

As shown in Figure 3, all the previous considerations cannot be applied to the curve obtained at *F* = 1 J/cm^2^, because such a fluence bring the target in the temperature regime $T∼0.9T_{c}$ where the phase-explosion phenomena occurs along with fast vaporization. The top-hat profile of this curve is given by the fact the surface temperature has been artificially set at $T∼0.9T_{c}$ in the simulation, since the explosive regime is not covered by our model. Even if phase-explosion is not present in this model, a useful information can be extracted, which is an estimate of the absorbed energy per unit area at which such an ablative regime occurs. This set of simulations estimate this value between *F* = 0.8 J/cm^2^ and *F* = 1 J/cm^2^.

**Figure 4:**
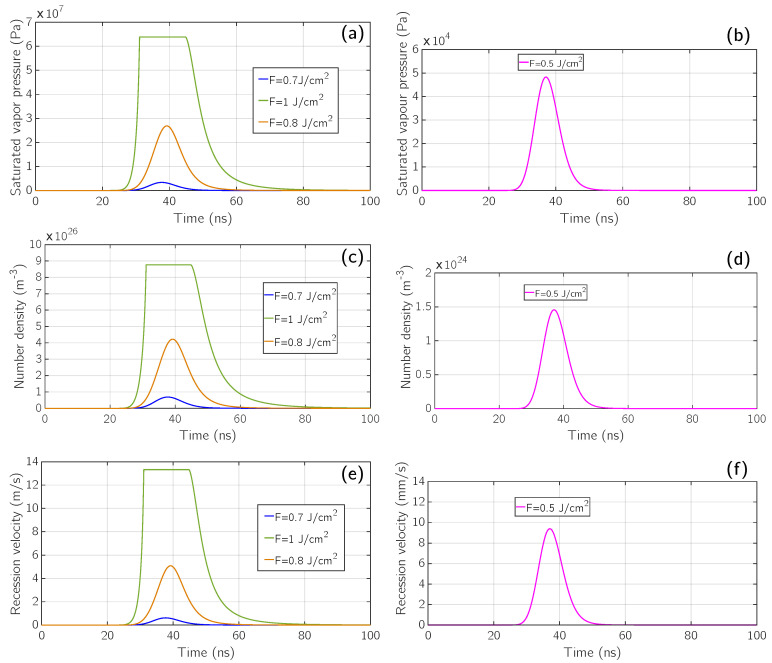
Saturation pressure (panels **a**,**b**), number density (panels **c**,**d**) and recession velocity (panels **e**,**f**) over time for different fluence values *F* = 0.7 J/cm^2^, 0.8 J/cm^2^ and 1 J/cm^2^ (blue, orange, green) and *F* = 0.5 J/cm^2^ (pink).

Now, by looking at Figure 4, panels (a) and (b), we see the plots of the saturated vapor pressure with respect to time for the previous fluences. In panels (a) and (b) all the four previous fluences are again considered, including the simulation at *F* = 1 J/cm^2^ in which phase explosion occurs. In this particular regime the maximum value of the saturated vapor pressure is from 600 atm to 700 atm. The behavior of these curves reflects the one of the surface temperature since they are computed making use of the Clausius-Clapeyron relation (9), along with the Watson relation [39].

According to (10) high values of pressure can only be obtained going well above the normal boiling point $T_{b}$. In fact, paying attention to Figure 4 panel (b), we see that the maximum value of the saturated vapor pressure for a fluence of 0.5 J/cm^2^ is three orders of magnitude less than the one for higher fluences, since its maximum value of surface temperature remains below the boiling temperature. This strong difference is also depicted in (a), where the pink curve is so low that it cannot be distinguished by the horizontal axis.

Moving to Figure 4 panels (c) and (d) we can observe the behavior of the atomic number density over time. Its behavior is analogous to the saturated vapor pressure since it was computed making use of the ideal gas law (11). In panel (c) its maximum value ranges from 8 × 10^26^ atoms/m^3^ to 9 × 10^26^ atoms/m^3^, while in panel (d) a loss of three orders of magnitude is again observed.

In Figure 4 panels (e) and (f), the surface recession velocity behavior due to fast vaporization is depicted. It was computed by making use of the continuity condition in Equation (14). In panel (e), it can be seen that the order of magnitude is m/s, and its top-hat value is close to 13 m/s. Looking at the far right part of the plot (80 ns ÷ 100 ns), it can be noticed that at this time the emission of material is practically over and, since we are mainly focused on the gas-dynamic evolution of the emitted material, we decided to stop our simulations at 100 ns. In panel (f) the recession velocity moves from m/s to mm/s.

**Figure 5:**
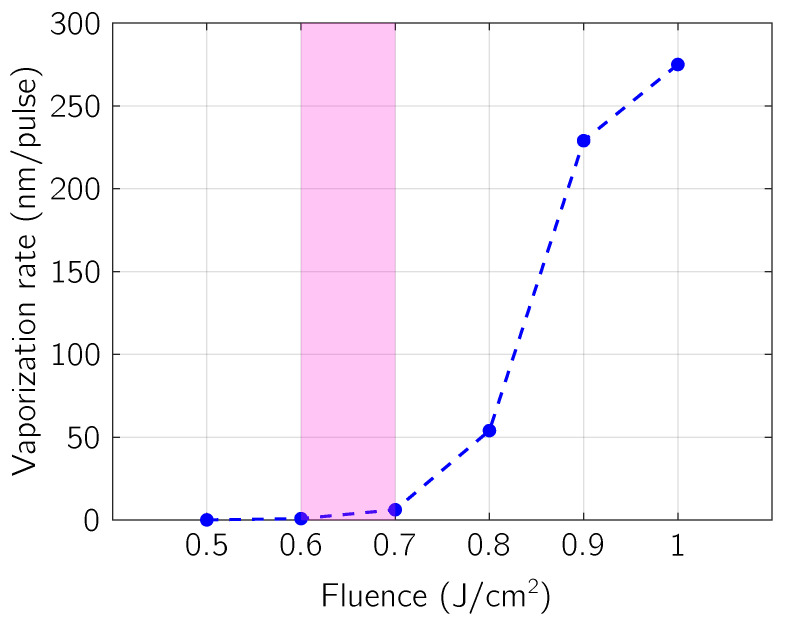
Vaporization rate as a function of fluence. The highlighted region represents the estimated vaporization threshold interval.

The time integration of functions reported in Figure 4 panels (e) and (f) gives average vaporization contribution to the ablation depth and it is depicted in Figure 5 as a function of the absorbed laser fluence. It can be seen that for values lower than 0.6 J/cm^2^ the ablative contribution of vaporization is negligible (ablated depth lower than 1 nm), while it grows for larger values of the absorbed fluence (up to hundreds of nm). This lets us estimate a theoretical absorbed fluence threshold for the vaporization process ($0.6\;J/cm{}^{2}<F_{\mathrm{th},\mathrm{vap}}<0.7\;J/cm{}^{2}$), i.e., the minimal energy density required to initiate material removal [45] by vaporization so that a surface ablation process can be detected. By looking at Figure 5 in connection with the curve at 0.5 J/cm^2^ in Figure 3, it follows that surface temperature not only has to reach $T_{m}$ but it also has to go beyond it so that vaporization gets relevance as ablative mechanism.

To estimate the total mass removed from the target by vaporization, the actual area of the laser spot must be multiplied by the total surface recession depth and by the target density.

**Figure 6:**
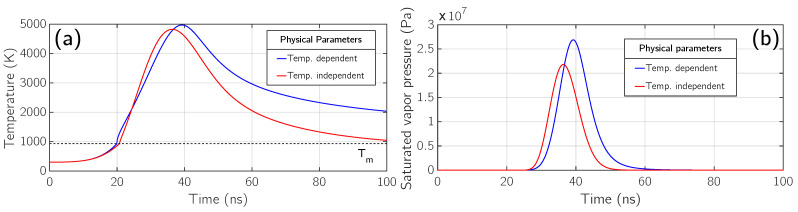
Comparison between simulations performed with temperature-independent (red) and temperature-dependent (blue) physical parameters at fluence F = 0.8 J/cm^2^. Panel (**a**): surface temperature. Panel (**b**): saturated vapor pressure.

In Figure 6, results of the model with a fluence *F* = 0.8 J/cm^2^ are shown considering both temperature-dependent (blue) and no temperature-dependent (red) physical parameters of aluminum. A comparison of surface temperature as a function of time in the two different situations is presented in panel (a) and two strong differences must be stressed here. First, the surface temperature maximum value is higher when temperature-dependent physical parameters are taken into account. Second, the surface cooling process is more effective if LA is modeled with constant parameters since it starts earlier and lowers the temperature more rapidly. In fact, at the end of the simulation ( 100 ns), surface temperature approaches the melting one ($T_{m}$) if no temperature-dependent parameters are considered, while it remains above 2000 K if temperature dependence is included in the model. This comparison shows how important is to consider temperature-dependent physical parameters when modeling LA if an estimate of the time at which resolidification occurs is a target of the simulation. This is of particular interest in all the applications of LA related to surface modification [46] and texturing [47], in which the dynamic of the melted surface plays a fundamental role.

As a consequence, the saturated vapor pressure exhibits different behaviours in the two situations, as depicted in Figure 6 panel (b). Its maximum value is lower with constant parameters and, in addition to that, it is worth noticing how reduced the amount of time is with the saturated vapor pressure of the order of tens of MPa in this situation. The value and the application time of the pressure exerted by the vapor on the surface is a fundamental quantity in LAP and here it is shown how strong is their connection with the temperature dependence of the physical parameters of the target.

**Figure 7:**
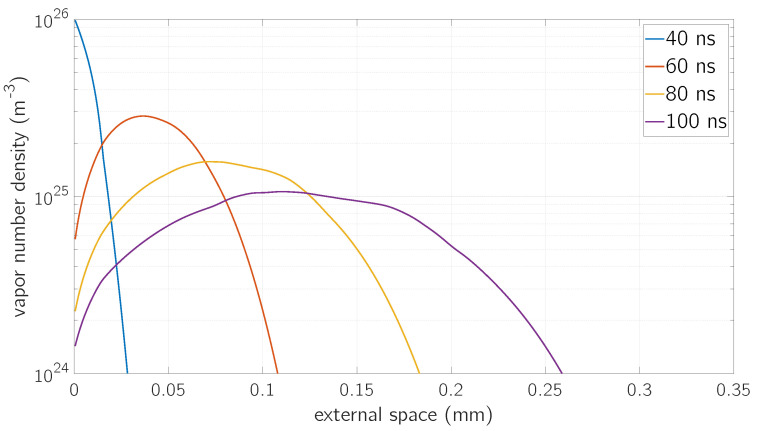
Atomic number density as a function of the distance from the surface at a fluence *F* = 0.8 J/cm^2^ for different time values.

In Figure 7, the number density of the outgoing vapor phase with respect to the external space is depicted at four different time instants: 40 ns, 60 ns, 80 ns and 100 ns. It represents the solution of the Euler’s Equations (15) with respect to the vapor density. The vapor expansion in the external vacuum region can be observed by looking at the maxima of the curves: in fact, such values move from left to right as time goes by.

Even if the full size of the external vacuum domain is set to 1 mm in the simulation, the region in which the change of vapor density can be clearly appreciated is lower than 0.3 mm in length for the simulation time-scale as it can be seen from the space domain of Figure 7.

**Figure 8:**
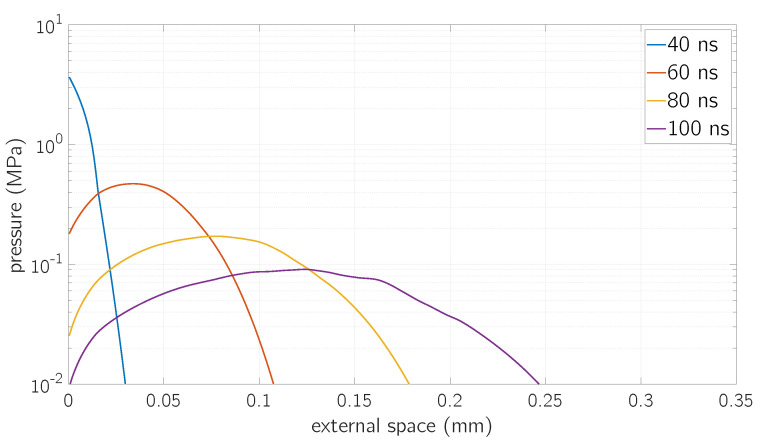
Values of vapor pressure as a function of the distance from the surface at a fluence *F* = 0.8 J/cm^2^ for different time values.

A similar behavior can be observed for the vapor pressure, depicted in Figure 8. This is due to the fact that the ideal gas law has been taken as a closure relation for the solution of the Euler equation.

**Figure 9:**
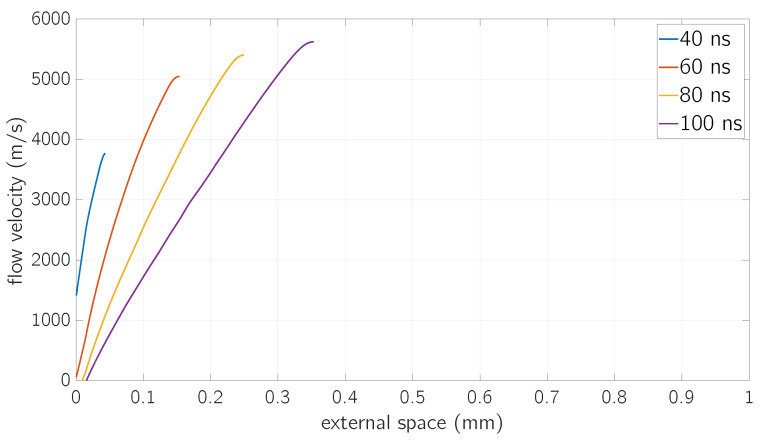
Values of flow velocity as a function of the distance from the surface at a fluence *F* = 0.8 J/cm^2^ for different time values.

The vapor expansion occurs with a flow velocity which depends on both space and time and it is shown in Figure 9. The position of such flow velocity front is correctly far away from the maximum values of vapor density since the mass conservation relation is included in the Euler Equations (15). The velocity of these fronts is monotonously increasing with time in our simulation, but this behaviour should come to an end since the momentum conservation relation is included in the Euler Equations (15). Regarding the increase in speed, both with time and distance from the surface, note that these trends are a genuine consequence of the equations of gas-dynamics [30,48]. Also shown in Figure 9, flow velocity exhibits a monotonously increasing linear behaviour, in agreement with other one-dimensional kinetic simulations [49].

### 5.1. Comparison with Experimental Results

First of all it, must be noticed that the order of magnitude of the computed flow velocity, in Figure 9, is ∼km/s. For a given time instant, it is a continuously increasing function of the external space, up to a sudden drop, which marks the end of the vapor expansion region.

The speed of the particles coming from the vaporization processes induced by laser pulses has been measured experimentally over many years [50,51] and is certainly consistent with the data we have calculated and reported. In particular, the experimental work by R. M. Gilgenbach et al. [29] is of great relevance since it was performed in the exact experimental conditions simulated in our work (excimer KrF laser with λ = 248 nm, FWHM = 20 ns, ablation in vacuum). Irradiating an aluminum target in vacuum with a laser fluence below the phase-explosion threshold, the reported experimental velocities are between 4.5 km/s and 6 km/s, in optimum agreement with our calculated results shown in Figure 9. The below threshold fluences considered in [29] are: 1.6 J/cm^2^, 2.2 J/cm^2^ and 2.6 J/cm^2^. As expected, they are a little higher than the ones considered here because, as experimentally proved by Wood et al. [51], plasma is formed by absorption of laser photons, thus reducing the actual fluence reaching the target (as considered in our work where indeed the reported fluence is the absorbed one).

The total amount of ablated material has been also widely investigated from an experimental point of view. In this field, J.M. Fishburn et al. [52] investigated the different mechanisms involved in crater formation in aluminum, under ns laser irradiation, being able to discriminate the actual role of vaporization among others (in particular melt displacement and phase explosion). For experimental conditions (λ = 511 nm, FWHM = 30 ns, ablation in vacuum) close to the ones considered here, and for fluences below the phase-explosion threshold, the depth of the crater dug by vaporization is of the order of a few tens of nm. Similar values are calculated with our model when the surface recession velocity, shown in Figure 4e and due to vaporization, is integrated in time (for example, the crater depth results of 54 nm for 0.8 J/cm^2^ of absorbed laser fluence). Moreover, the vaporization ablation threshold measured in [52] is reported to be 1.5 J/cm^2^, slightly higher (considering the experimental uncertainties) than the absorbed fluence interval shown in Figure 5, but easily explained by plasma-shielding effect as previously discussed.

Finally, the experimental recoil momentum induced by ablation can be considered for comparison. In fact, in Figure 7 of [52], in the case of vaporization-dominated LA (F < 3.2 J/cm^2^), the measured recoil momenta are reported. The recoil momentum ($\Delta p_{\mathrm{teo}}$), in our model, may be written as:

(33) $$\Delta p_{\mathrm{teo}}≃\int_{0}^{\tilde{t}}Ap_{\mathrm{rec}}\left(t\right)dt,$$

where *A* is the laser spot area, $\tilde{t}$ is a later time when vaporization becomes negligible, and $p_{\mathrm{rec}}=0.55p_{s}$ is the recoil pressure according to [20]. Our recoil momentum results to be $\Delta p_{\mathrm{teo}}$ = 2.1 nNs when *F* = 0.8 J/cm^2^, which is in very good agreement with the experimental data of [52].

As a final remark, we would to point out that any experimental data obtained from LA of aluminum could be affected by the presence of a thin film of its oxide (${\mathrm{Al}}_{2}O_{3}$) on pure Al target surface, unless experimental precautions are taken which guarantee no contamination inside the ablation chamber, as both theoretically and experimentally discussed in [53].

## 6. Conclusions

In this work, we presented a 1-D continuum model for ns-LA ablation in a vacuum, of aluminum, for absorbed laser fluence in the range 0.5 ÷ 1 J/cm^2^, with temperature-dependent thermophysical, optical, and transport properties. The thermal model is coupled to the gas-dynamical evolution of the expanding vapor.

We restricted ourselves to an ablative regime where vaporization is the leading mass removal process and we showed how strongly important is to consider temperature-dependent physical parameters to compute the actual vapor pressure exerted by the vapor on the surface, which is a fundamental value, for example, in the context of LAP.

We resolved the actual vapor expansion in the first 100 ns in both space and time, making a computational step forward with respect to the present literature [22], where no gas-dynamical processes were quantitatively included in the model describing laser ablation.

We developed a robust numerical scheme to take care of the solution of Euler equations with time-dependent boundary conditions which permitted the comparison between temperature-dependent and constant parameters, thus proving that the former are necessary to properly study the melting/resolidification process and the surface dynamics in general.

Our calculated expansion velocities are of the order of ∼km/s and are in strong agreement with the experimental measurements of R.M. Gilgenbach et al. [29] using laser parameters very similar to ours. In addition, the experimental data regarding crater depth, ablated mass, and recoil momentum in laser irradiated aluminum target, as reported by J.M. Fishburn et al. [52] for laser pulse conditions similar to ours, compare very well with the results of our model.

## Figures and Tables

**Table 1:** Aluminum thermophysical data.

Parameter	Symbol	Value	Unit
Melting point	$T_{m}$	933	K
Boiling Point (at $P_{\mathrm{atm}}$)	$T_{b}$	2743	K
Critical temperature	$T_{c}$	6319	K
Critical density	$\rho_{c}$	634	$\mathrm{kg}/m^{3}$
Latent heat of melting	$\Delta h_{m}$	$1.71\times 10^{4}$	J/mol
Latent heat of vaporization	$\Delta h_{v}$	$2.84\times 10^{5}$	J/mol

**Table 2:** Aluminum critical coefficients.

Parameter	Value
$\alpha_{c}$	0.109
$\beta_{c}$	0.325
$\Delta_{c}$	0.51
$D_{0}$	1.1
$D_{1}$	−0.17
$C_{1}$	1.75
$C_{2}$	0.08

**Table 3:** Drude two-critical point model parameters.

$\epsilon_{\infty }$		1.0	
$A_{1}$	5.2306	$A_{2}$	5.2704
$\phi_{1}$	−0.51202	$\phi_{2}$	0.42503
$\Omega_{1}$	$2.2694\times 10^{15}$	$\Omega_{2}$	$2.4668\times 10^{15}$
$\Gamma_{1}$	$3.286\times 10^{14}$	$\Gamma_{2}$	$1.7731\times 10^{15}$

## Abbreviations

The following abbreviations are used in this manuscript:

CFL	Courant-Friedrich-Levy
FTCS	Forward Time Centered Space
FV	Finite Volumes
FWHM	Full Width Half Maximum
KL	Knudsen Layer
LA	Laser Ablation
LAP	Laser Ablation Propulsion
LIBS	Laser Induced Breakdown Spectroscopy
LTP	Low Temperature Plasma
PLD	Pulsed Laser Deposition
UAE	Unsteady Adiabatic Expansion
